# Hippocampal CA3 transcriptional modules associated with granule cell alterations and cognitive impairment in refractory mesial temporal lobe epilepsy patients

**DOI:** 10.1038/s41598-021-89802-3

**Published:** 2021-05-13

**Authors:** Silvia Yumi Bando, Fernanda Bernardi Bertonha, Luciana Ramalho Pimentel-Silva, João Gabriel Mansano de Oliveira, Marco Antonio Duarte Carneiro, Mariana Hiromi Manoel Oku, Hung-Tzu Wen, Luiz Henrique Martins Castro, Carlos Alberto Moreira-Filho

**Affiliations:** 1 Department of Pediatrics, Faculdade de Medicina da Universidade de São Paulo, São Paulo, SP 05403-900 Brazil; 2 Department of Neurology, Faculdade de Ciências Médicas da Universidade Estadual de Campinas, UNICAMP, Campinas, SP 13083-887 Brazil; 3 Department of Neurology, Faculdade de Medicina da Universidade de São Paulo, São Paulo, SP 05403-900 Brazil; 4 Epilepsy Surgery Group, Hospital das Clínicas da FMUSP, São Paulo, SP 05403-900 Brazil

**Keywords:** Neurology, Transcriptomics

## Abstract

In about a third of the patients with epilepsy the seizures are not drug-controlled. The current limitation of the antiepileptic drug therapy derives from an insufficient understanding of epilepsy pathophysiology. In order to overcome this situation, it is necessary to consider epilepsy as a disturbed network of interactions, instead of just looking for changes in single molecular components. Here, we studied CA3 transcriptional signatures and dentate gyrus histopathologic alterations in hippocampal explants surgically obtained from 57 RMTLE patients submitted to corticoamygdalohippocampectomy. By adopting a systems biology approach, integrating clinical, histopathological, and transcriptomic data (weighted gene co-expression network analysis), we were able to identify transcriptional modules highly correlated with age of disease onset, cognitive dysfunctions, and granule cell alterations. The enrichment analysis of transcriptional modules and the functional characterization of the highly connected genes in each trait-correlated module allowed us to unveil the modules’ main biological functions, paving the way for further investigations on their roles in RMTLE pathophysiology. Moreover, we found 15 genes with high gene significance values which have the potential to become novel biomarkers and/or therapeutic targets in RMTLE.

## Introduction

Epilepsy affects 50 million people worldwide and is characterized by unprovoked recurrent seizures due to abnormal neuronal discharge, and by the neurobiological, cognitive, and psychological consequences of seizure recurrence^1^. Mesial temporal lobe epilepsy (MTLE), the commonest focal epilepsy in adults, involves the medial structures of the temporal lobe and hippocampal sclerosis (HS) constitutes its most frequent pathological abnormality^2^. MTLE is often associated with a history of prolonged febrile seizures in early childhood or other initial precipitating injuries (IPI)^3^. About 40% of the MTLE patients with a history of febrile seizures develop refractory (drug-resistant) epilepsy (RMTLE)^4^. Furthermore, early IPI and early epilepsy onset (i.e., in the first years of life) are frequently associated respectively with severe hippocampal neuronal loss^5^ and refractory epilepsy^6^. Refractory epilepsy increases the risk of psychosocial dysfunction, cognitive decline, and sudden unexpected death of patients^7^.

RMTLE patients can benefit from surgical treatment^8^, and hippocampal explants obtained at epilepsy surgery are a valuable material for investigating the cellular, molecular and genomic mechanisms underlying refractory epilepsy. Studies in surgical specimens helped to unveil the relevance of hippocampal areas with regard to the temporal lobe epilepsies (TLE) progression and, particularly, of the dentate gyrus (DG) in biological processes related to early and late disease stages^9^. Pathomorphological studies showed compromised neurogenesis and significant DG cell loss in RMTLE patients^10,11^ and in vitro and animal model assays showed that inadequate circuit control of DG-CA3 synapses leads to epilepsy, since DG acts as a “gate”, protecting hippocampal circuits from overexcitation^12,13^. These observations on DG-CA3 transition dynamics were also supported by in vitro studies of living networks of DG and CA3 neurons^14^, and by computational simulation studies of the DG-CA3 neuronal network^15^. Furthermore, multi-omics analyses performed in the pilocarpine rat model of MTLE suggested enhanced epileptogenesis in the CA3 region when compared to the DG, with most of the transcriptional and protein expression alterations occurring in CA3^16^. Additionally, the integrative study of CA3 transcriptional signatures and DG histopathology in hippocampal explants surgically obtained from RMTLE patients allowed our group, and others, to portrait the disease as a disturbed network of gene–gene interactions^17–21^.

On the other hand, even considering the good results attained by the surgical treatment for refractory epilepsy, only a minority of patients with RMTLE are ever referred to epilepsy surgery, and often too late to prevent serious disabilities^22^. This situation reinforces the demand for identifying genomic mechanisms that can be targeted for novel preventive and drug-based therapeutic interventions^23^. The transcriptome analysis pipeline for this task usually involves building gene co-expression networks, finding and functionally characterizing the transcriptional modules correlated to particular traits, their hub genes, which are associated to specific cellular processes or link different biological processes, and their genes significantly correlated with a particular trait^24–26^. As pointed out by Gaiteri et al.^26^, “changes in gene–gene correlation may occur in the absence of differential expression, meaning that a gene may undergo changes in regulatory pattern that would be undetected by traditional differential expression analysis”. Here, by using that pipeline and integrating demographic, clinical, cognitive, histopathological and genomic data, we investigated: (i) if in patients with RMTLE the cognitive function impairments and DG histological alterations could be correlated with CA3 transcriptional modules; (ii) if the genes with high connectivity (hubs) or high gene significance in the trait-correlated modules were related to epilepsy-relevant cellular and molecular mechanisms.

## Results

**Figure 1:**
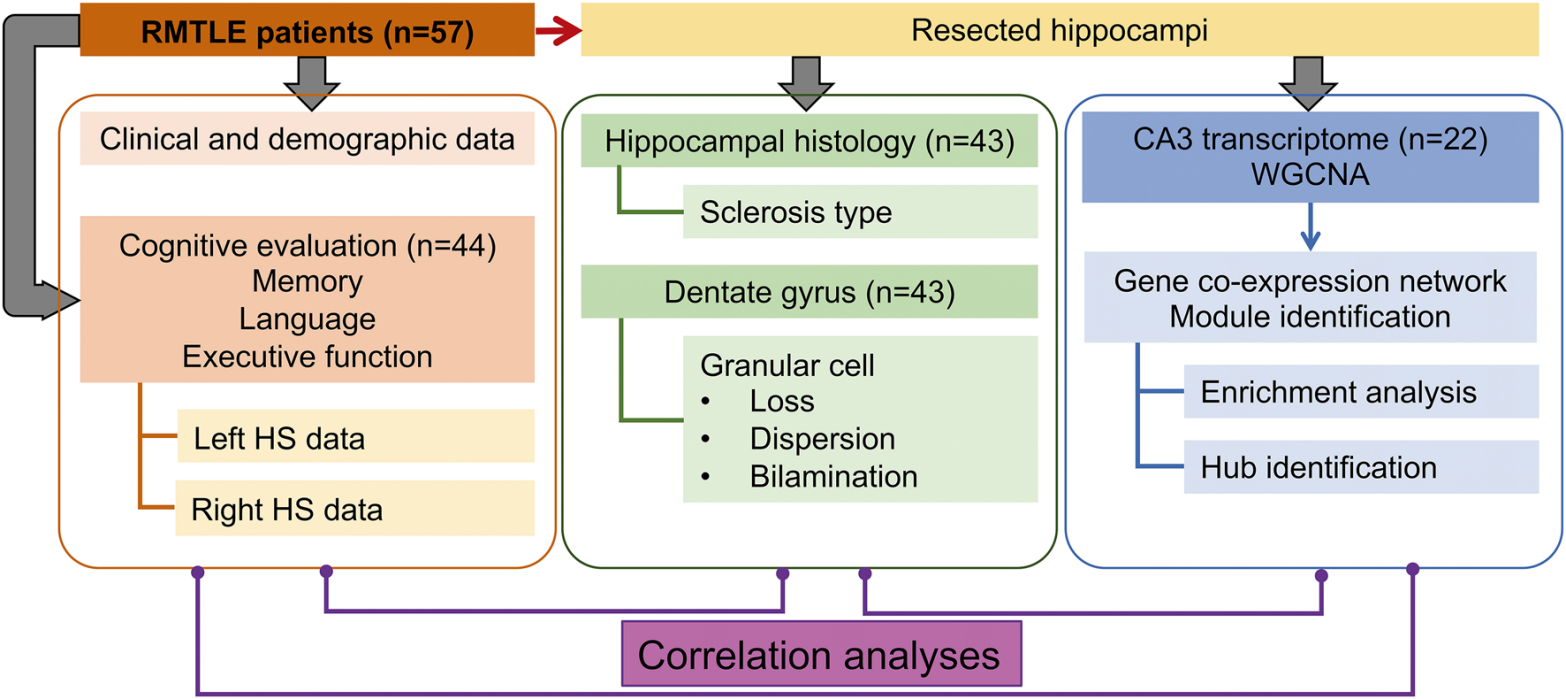
Study Workflow. Integrative analysis of demographic, clinical, histopathological, and genomic data.

We studied 57 RMTLE patients with unilateral mesial temporal sclerosis (MTS) who had been submitted to corticoamygdalohippocampectomy (Supplementary Table S1 online). Patients’ cognitive function was assessed preoperatively. Histological analysis was performed in the resected hippocampal specimens to diagnose and to determine the pattern of hippocampal sclerosis (HS) and to assess the grades of cell loss, dispersion and bilamination. CA3 tissue explants for genomic studies were obtained during epilepsy surgery. Weighted Gene Co-expression Network Analysis (WGCNA)^24^ was used for constructing the module-trait relationships considering demographic, clinical, cognitive, histopathologic and genomic data (Fig. 1).

### Cognitive function evaluation

Forty-four RMTLE patients and two control groups totaling 60 normal healthy volunteers—one control subgroup for memory and executive function evaluation (n = 40), and another for language evaluation (n = 20)—were matched for age, gender, education, and IQ scores (Supplementary Table S2 online). Patients and controls underwent a cognitive test battery for language, memory, and executive function (Supplementary Table S3 online). The patient’s group was divided into two subgroups: (i) LHS subgroup (n = 27), encompassing left HS patients (ii) RHS subgroup (n = 17) for right HS patients. Cognitive tests score from patients and controls were submitted to ROC analysis for identifying classificatory tests and to obtain the corresponding cut-off. Additionally, we compared patient’s vs. controls’ performance on individual tests for memory, language and executive function (Supplementary Table S3 online). The LHS subgroup showed impaired performance (*P* < 0.05) in semantic and phonological fluency, verbs and proper noun naming, RAVLT total and late recall, logical memory, Rey-Osterrieth Complex Figure (ROCF), Stroop test, and Phonemic Fluency (FAS) tests (Supplementary Table S3 online). The RHS subgroup showed impaired performance relative to control group (*P* < 0.05) in semantic fluency, object, verbs and proper noun, and responsive naming, RAVLT immediate recall and delayed recognition subsets, RVDLT and facial recognition, ROCF, Stroop test, and FAS tests (Supplementary Table S3 online). These results were used for scoring failures in memory, language, and executive function impairment (Supplementary Table S4 online). Patients were then classified in two subgroups according to cognitive impairment: severe, where total score > average score, or mild, where total score ≤ average score (Supplementary Tables S1 and S4 online). Fisher’s exact test for severe cognitive impairment did not show significant probability (*P* > 0.05) for laterality.

### Histopathology

Forty-three resected hippocampi were used for histopathological analysis (Supplementary Table S1 online). The HS types were classified according to Blümcke et al.^27^. Semiquantitative assessment was performed on dentate gyrus abnormalities, particularly cytoarchitectural disorganization: granule cell loss (GCL), dispersion (GCD), and bilamination (GCB). Table 1 shows the frequency distribution of histological features. HS ILAE type 1 (severe CA1 and CA4 neuronal loss and gliosis) was the most frequent type (90.9%), followed by HS ILAE types 2 (CA1 neuronal loss and gliosis), and one no-HS (preserved cell density and gliosis only). GCL was observed in 42 samples, being GCL grade 2 the most frequent type (41.9%) followed by grades 3 and 0–1. GCD was observed in 38 samples and the most frequent type was GCD grade 3 (37.2%), followed by grades 1, 2 and 0. GCB was present in 15 out of 43 samples (34.9%).

**Table 1:** Demographic and clinical characteristics of 43 RTMLE patients and histological features of the patients' hippocampal samples.

Phenotypic characteristics		Number	%
Gender	Male	21	48.8
	Female	22	51.2
Severe memory impairment	Left HS (n = 18)	7	38.9
	Right HS (n = 12)	6	50.0
Severe language impairment	Left HS (n = 18)	5	27.8
	Right HS (n = 12)	5	41.7
Severe executive function impairment	Left HS (n = 17)	7	35.3
	Right HS (n = 12)	6	50.0
Brain side affected by HS	Left	25	58.1
	Right	18	41.9
IPI status	Febrile	15	34.9
	Afebrile	27	62.8
Age at IPI	Early (≤ 5 yrs)	25	58.1
	> 5 yrs	18	41.9
Age at disease onset	Early (≤ 5 yrs)	8	18.6
	> 5 yrs	35	81.4
HS ILAE type	1	40	90.9
	2	3	6.8
	No-HS	1	2.3
Grade of GCL	0 to 1	11	25.6
	2	18	41.9
	3	14	32.6
Grade of GCD	0	5	11.7
	1	13	30.2
	2	9	20.9
	3	16	37.2
Presence of GCB in the dentate gyrus	Yes	15	34.9
	No	28	65.1

### Clinicopathological correlation analyses

Correlation analyses for the LHS and RHS subgroups were performed between pairs of selected demographical, clinical and pathological variables: gender, age at surgery, age at IPI, age of epilepsy onset, cognition (memory, language, and executive function) and histological features (HS type, grade of GCL or GCD, and presence of GCB). A statistically significant and positive correlation was found for GCL and memory impairment grades (r = 0.47, *P* = 0.02) in the LHS subgroup.

### Weighted gene co-expression network analysis

**Figure 2:**
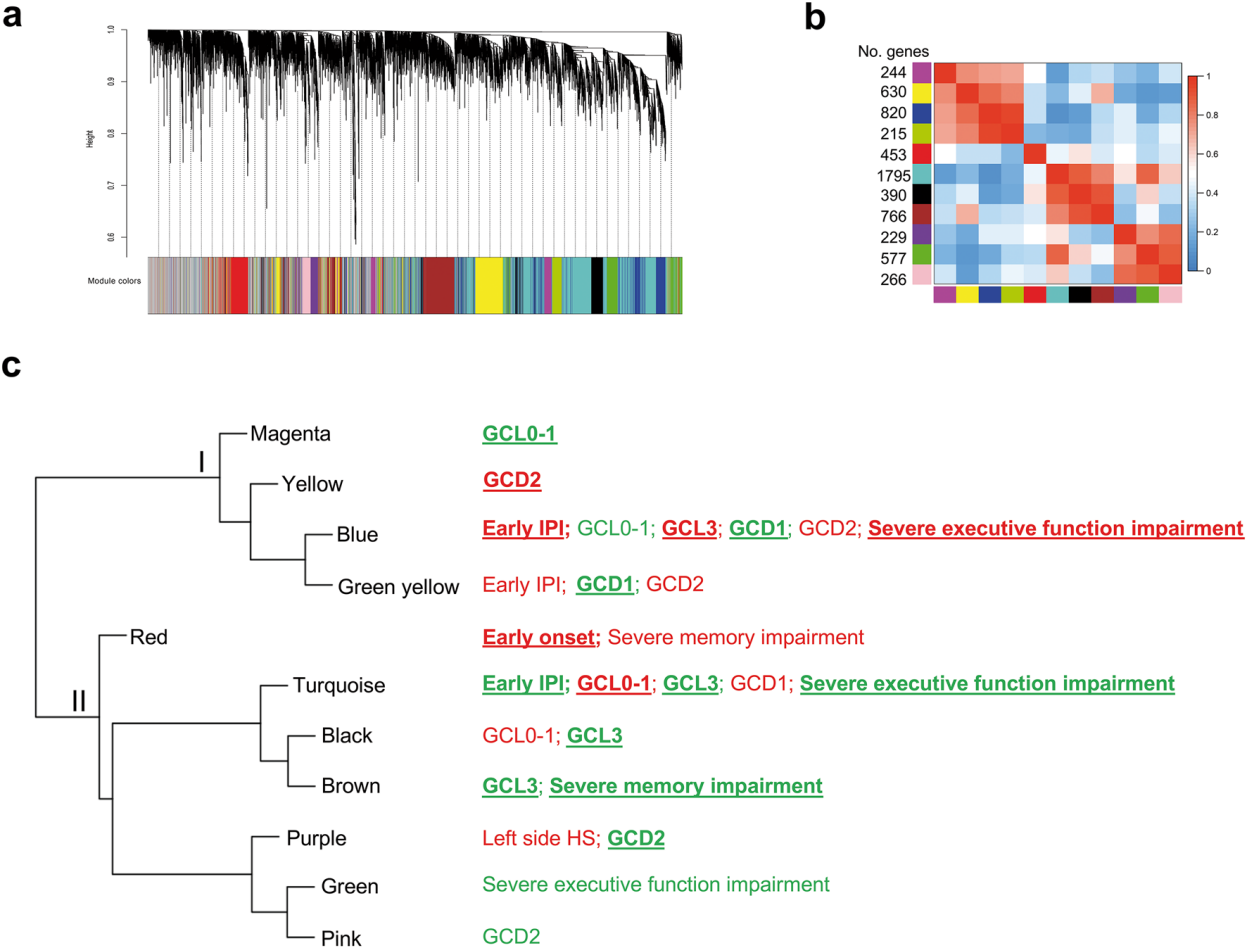
WGCNA analysis. Gene dendrogram and gene clustering analysis for module identification (**a**). Heatmap plot of the adjacencies in the eigengene network (**b**). Hierarchical clustering dendrogram of module eigengenes-evidencing the presence of two meta-modules (I and II)-and traits significantly correlated with modules (**c**). Positive or negative module-trait correlations depicted in red or green, respectively; the high module-trait correlations (r >|0.50|) are indicated by bold underlined letters. Network was constructed using the WGCNA package^24^ (version 1.69-81) in R environment (version 3.4.4^93^; https://horvath.genetics.ucla.edu/html/CoexpressionNetwork/Rpackages/WGCNA/).

Total RNA samples were obtained from the hippocampal CA3 region of 22 RMTLE patients (Supplementary Table S1 online) and used for DNA microarray hybridizations. The normalized gene expression data of 8,104 GO (Gene Ontology) annotated genes were used for network construction and module identification by WGCNA. Pearson’s correlation coefficient was used for obtaining gene co-expression network and dynamic tree cut algorithm was used for dendrogram`s branch selection for module identification. Eleven transcriptional modules were identified. The module sizes ranged from 215 (green yellow module) to 1,795 (turquoise module) genes (Fig. 2a,b). The resulting eigengene network presented two meta-modules, here named I and II, as displayed in Fig. 2c.

#### Module-trait correlation analyses

We performed two module-trait correlation analyses: one for demographic, clinical, and histopathological traits including all patients in LHS and RHS subgroups and another for cognitive function, conducted separately for LHS and RHS subgroups (due to the significant correlation between GCL and severe memory impairment in LHS). A statistically significant module-trait correlation was found only for LHS patients. All transcriptional modules presented significant correlation (*P* < 0.05) with at least one trait and nine of these modules-four in the meta-module I and five in the meta-module II—showed high correlation values (r >|0.50|) with at least one trait, as described (Fig. 2c; Supplementary Figs. S1 and S2 online). In the meta-module I the blue module is positively correlated with early IPI (E-IPI), granule cell alterations (GCL grade 3), and severe executive function impairment (SEFI), whereas in the meta-module II the turquoise module is negatively correlated with those three traits. The yellow module in the meta-module I is positively correlated with GCD2. Inversely, the purple module in meta-module II is negatively correlated with that trait. The red module-in a separate branch in the meta-module II (Fig. 2c)-is positively correlated with early disease onset (E-onset). Interestingly, the meta-module I harbors modules positively correlated with severe phenotypes (GCL3, GCD2, E-IPI, SEFI) and negatively correlated with mild phenotypes (GCL0-1, GCD1), whereas in meta-module II these correlations are quite inverted.

#### Functional characterization of modules

**Figure 3:**
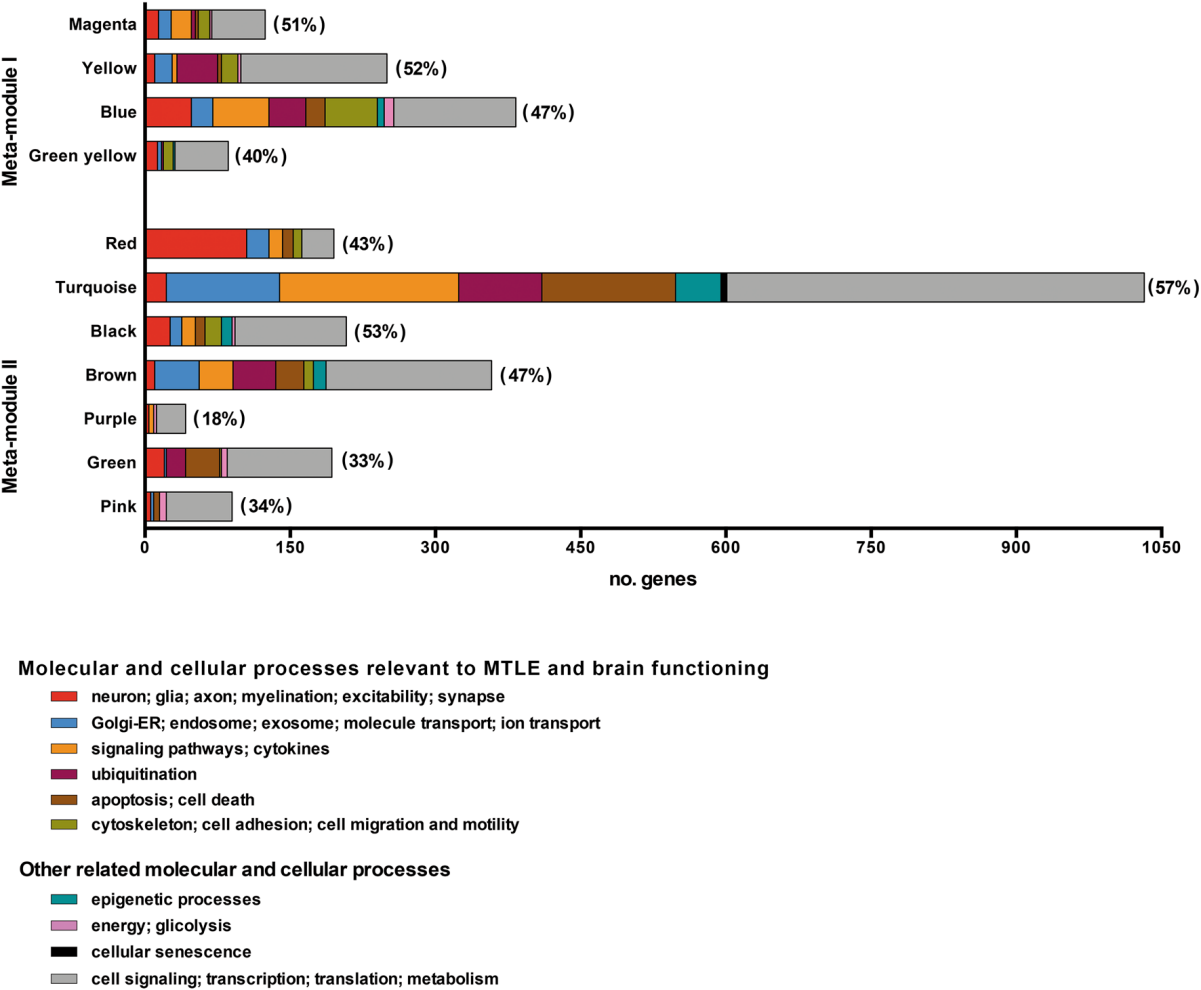
Histogram of enriched GO Biological Process (BP) terms for each WGCNA gene module. Different colors represent GO BP terms comprised in two main categories: (i) molecular and cellular processes relevant to MTLE and brain functioning; (ii) other related molecular and cellular processes. Between round brackets: percentage of genes over-represented in BP terms.

Transcriptional modules often represent biological processes that can be phenotype specific^28^. Functional enrichment among the genes within a module is widely used for disclosing its biological meaning^28^. We used a web-based GO enrichment analysis tool^29^ and found that between 18 and 57% of the genes within the modules were highly correlated with at least one trait were significantly (*P* < 0.05) over-represented in GO biological processes (BP) terms (Fig. 3). These terms were arbitrarily grouped in two major functional categories: (i) molecular and cellular processes relevant to MTLE and brain functioning (ii) other related molecular and cellular processes (Fig. 3, Supplementary Table S5 online). The complete list of the BP terms found for all modules is presented in Supplementary Datasheet S1 online. Three transcriptional modules-red, turquoise and blue-have 30 percent or more of their genes over-represented in the category “molecular and cellular processes relevant to MTLE and brain functioning” (Fig. 3; Supplementary Table S5 online). Noteworthy, the blue and turquoise modules are associated with more traits than any of the other modules (Fig. 2c), and the red module—highly and positively correlated with E-onset—showed 163 genes associated to processes linked to MTLE, of which 105 (Supplementary Table S5 online) are related with neuron/excitability processes (neuron, glia, axon, myelination, excitability, and synapse).

#### Categorization of high-hierarchy (HH) genes

**Figure 4:**
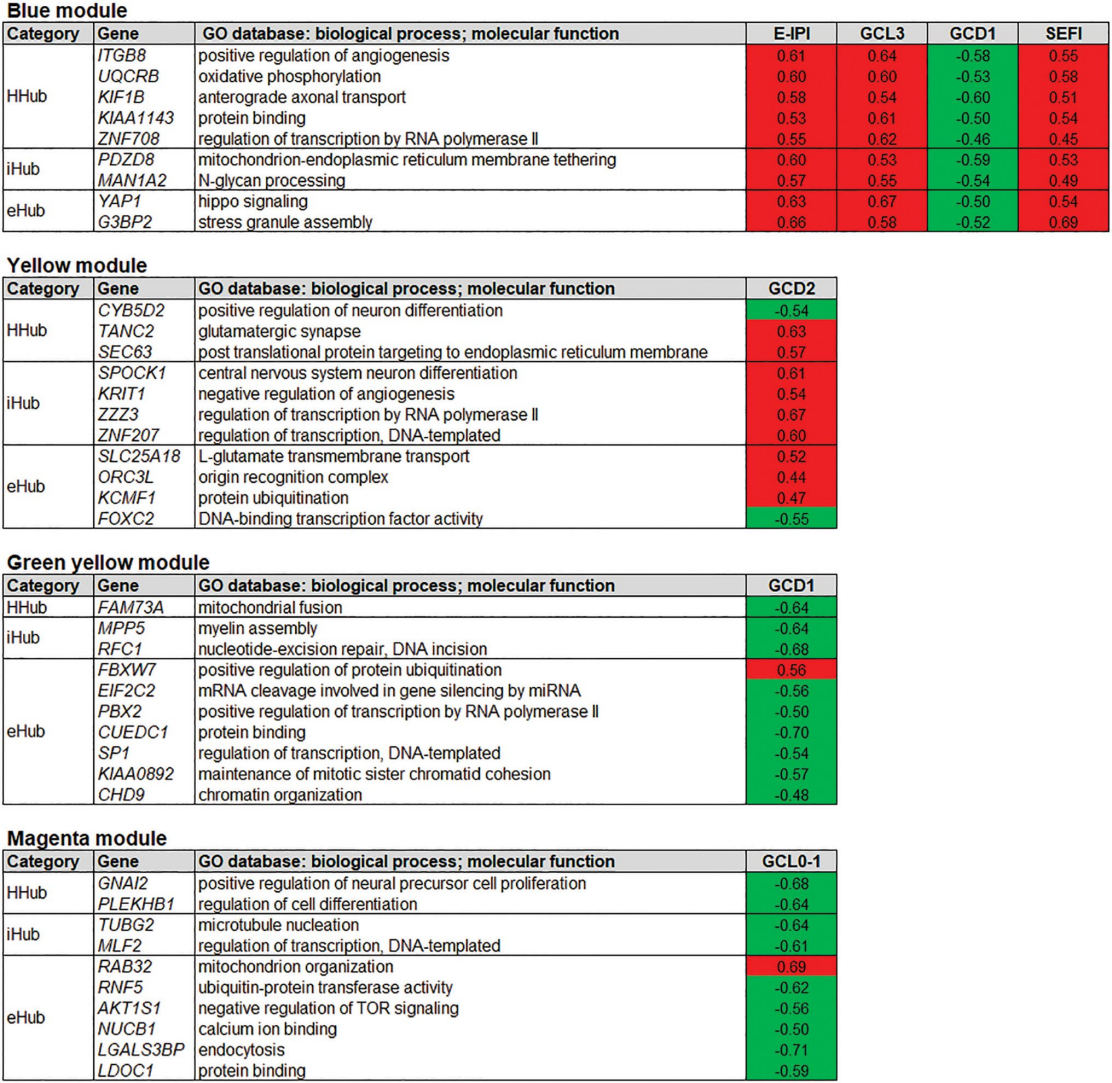
High hierarchical genes (Hhubs, iHubs, and eHubs) of the meta-module I present in modules highly associated with at least one trait. Each HH gene is identified by its hierarchical categorization and GO biological process or molecular function. Columns in red or in green indicate genes with positive (i.e. hyper-expressed) or negative (i.e. hypo-expressed) GS values for a specific trait, respectively. NS indicates gene with non-significant (*P* ≥ 0.05) GS value for the specific trait. E-IPI stands for early IPI. SEFI stands for severe executive function impairment. The abbreviations-GCD1, GCD2, GCL0-1, GCL3, HS ILAE type 3-indicate histopathological alterations, as described in the Results and Material and Methods sections.

**Figure 5:**
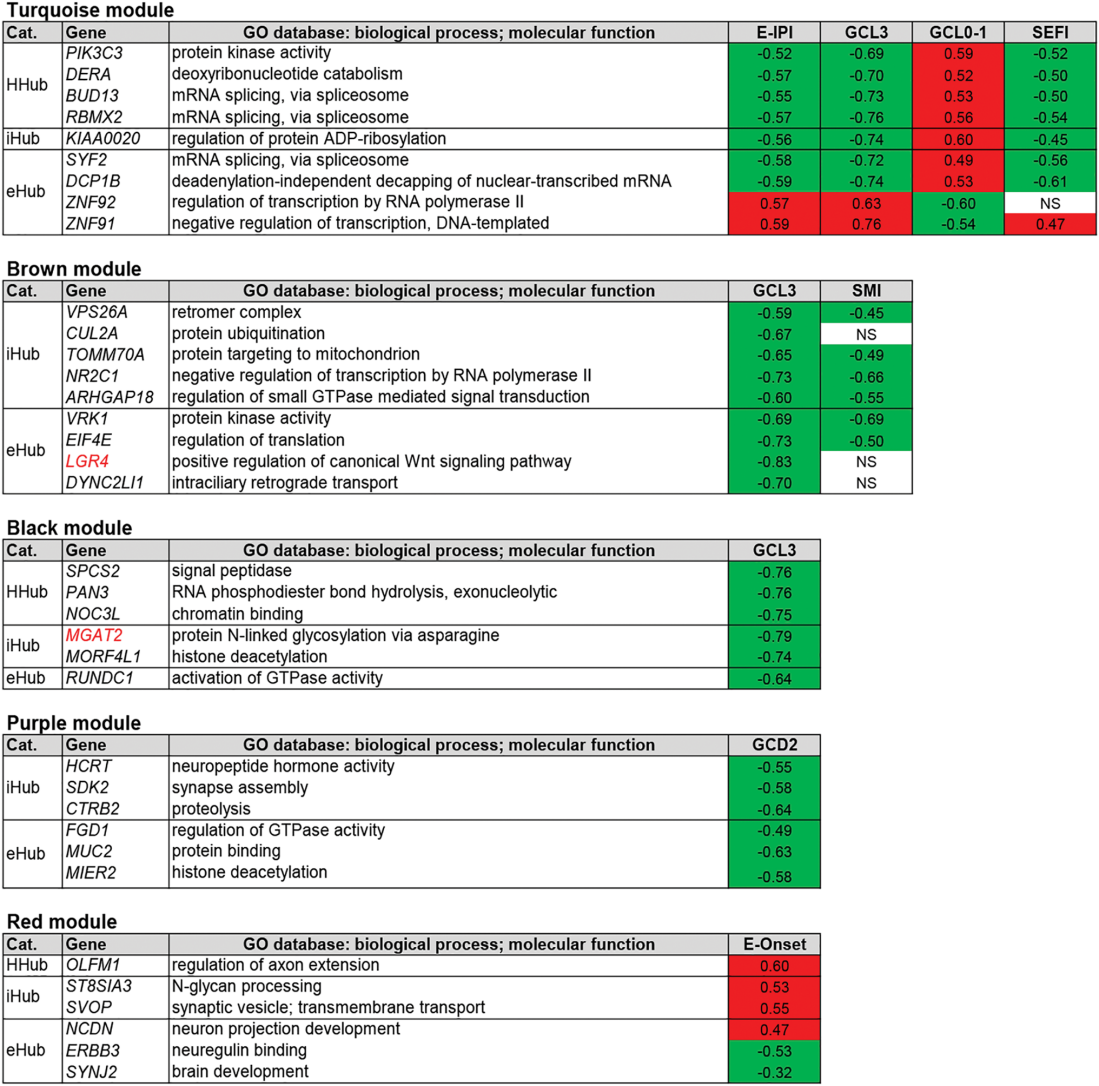
High hierarchy genes (Hhubs, iHubs, and eHubs) of the meta-module II present in modules highly associated with at least one trait. Each HH gene is identified by its hierarchical categorization and GO biological process or molecular function. Genes that are HH and HGS (see Table 2) appear in red lettering. Columns in red or in green indicate genes with positive (i.e. hyper-expressed) or negative (i.e. hypo-expressed) GS values for a specific trait, respectively. NS indicates gene with non-significant (*P* ≥ 0.05) GS value for the specific trait. E-IPI stands for early IPI; E-Onset stands for early onset; SEFI stands for severe executive function impairment; SMI stands for severe memory impairment. The abbreviations-GCD2, GCL0-1, GCL3-refer to histopathological alterations, as described in the Results and Material and Methods sections.

Highly connected genes hold the whole transcriptional network together and are either associated to specific cellular processes or link different biological processes^25^. Thus, we used connectivity measures for the hierarchical categorization of genes (see Methods) in the modules highly correlated with at least one trait^24^. HH genes were classified in one of three hierarchical categories according to the number of gene–gene links: (i) intramodular hub (iHub), a gene highly connected with other genes in the same module; (ii) high hub (Hhub), a hub that also has a high whole network connectivity; and (iii) intermodular hubs (eHub), a gene with low number of intramodular links but high whole network connectivity. Additionally, the gene significance (GS) value and the p-value obtained for each HH gene-trait correlation were used for obtaining the gene expression profiles that were positively (hyper-expression) or negatively (hypo-expression) correlated with histological and/or cognitive traits. A total of 76 HH genes were thereby found and categorized (Supplementary Figs. S3 and S4 online), being distributed into nine modules, as depicted in Figs. 4 and 5, corresponding to meta-modules I and II, respectively.

#### Functional interpretation of HH genes in trait-associated modules

The HH genes were interpreted in the context of the two eigengene network meta-modules. The meta-modules-identifiable as branches in the eigengene dendrogram—are sets of modules and genes with stronger relationship and reveal a higher order organization among gene co-expression modules^30^. Meta-modules are biologically significant^24^ and the functional enrichment analysis of their modules may help to reveal their meaning (Fig. 3). In Figs. 4 and 5, related to meta-modules I and II, respectively, the expression value profile of the HH genes corresponds, in a large measure, to the module-trait correlations above-mentioned (Fig. 2c).

#### HH genes in the meta-module I

In the blue module all HH genes are positively correlated with E-IPI, GCL3 and SEFI (Fig. 4) and two of its Hhubs, *ITGB8* and *UQCRB*, are involved in brain angiogenesis^31^ and in angiogenesis induction^32^, respectively. This is rather expectable since dysregulated angiogenesis and vascular remodeling are hallmarks of the epileptic brain^33^. Two other blue module genes, the Hhub *KIF1B* and the eHub *YAP1*, were shown to be essential for survival of hippocampal neurons and their hyper-expression in the sclerotic hippocampi may well constitute a compensatory mechanism^34,35^.

In the yellow module nine out of the eleven HH genes are positively correlated with GCD2 (Fig. 4). The HHub *TANC2* and the eHub *SLC25A18*, both hyper-expressed, belong to the glutamatergic excitatory system^36,37^, whose dysfunction leads to hyperexcitatory neural networks and neurotoxicity^38^. The iHub *SPOCK1*, also hyper-expressed, encodes the proteoglycan testican, which is expressed in hippocampal pyramidal neurons and has a functional role in reactive gliosis^39^. Reactive gliosis is a quite common finding in drug resistant TLE^27^. Noteworthy, the Hhub *CYB5D2*, that codes for neuferricin and is involved in hippocampal neurogenesis^40^, is negatively correlated with GCD2.

In the green yellow module the only gene positively correlated with GCD1 is the eHub *FBXW7*, which exerts a protective role against glutamate receptor-mediated excitotoxicity^41^. Finally, in the magenta module, all but one HH genes are negatively correlated with GCL0-1, the exception being the eHub *RAB32* whose upregulation is a marker for neuroinflammatory lesions^42^.

#### HH genes in the meta-module II

In this meta-module five out of seven modules harbor HH genes. The turquoise module is the largest in number of genes (Fig. 2b) and its module-trait correlation is almost the inverse of that found for the blue module in meta-module I (Fig. 2c). Most of the HH genes in this module are positively correlated with the mild phenotype GCL0-1 but negatively correlated with the severe phenotypes E-IPI, GCL3, and SEFI (Fig. 5). Two genes are indeed exemplary in this context: *PIK3C3*, essential for CNS neuronal homeostasis^43^ and for axon growth in hippocampal neurons^44^, and *DCP1B*, a potential regulator of human memory performance through its interaction with miR-138-5p^45^, are both negatively correlated with GCL3 and SEFI.

In the brown module all HH genes are negatively correlated with GCL3 and SMI (Fig. 5). This module harbors the eHubs *LGR4* (also an HGS gene), a positive regulator of the canonical Wnt/β catenin signaling pathway^46^ and *EIF4E*, a mTOR downstream effector^47^. The Wnt/β pathway is a principal positive regulator of adult hippocampal neurogenesis and its disruption contributes to the functional and structural of temporal lobe epilepsy abnormalities^48^. Conversely, an increased mTOR activation was observed in the sclerotic hippocampi from RMTLE patients^49^ and *EIF4E* was recently identified as therapeutic target in medically refractory epilepsy^50^. Here we found that *LGR4* and *EIFE4* were both negatively correlated with GCL3.

**Figure 6:**
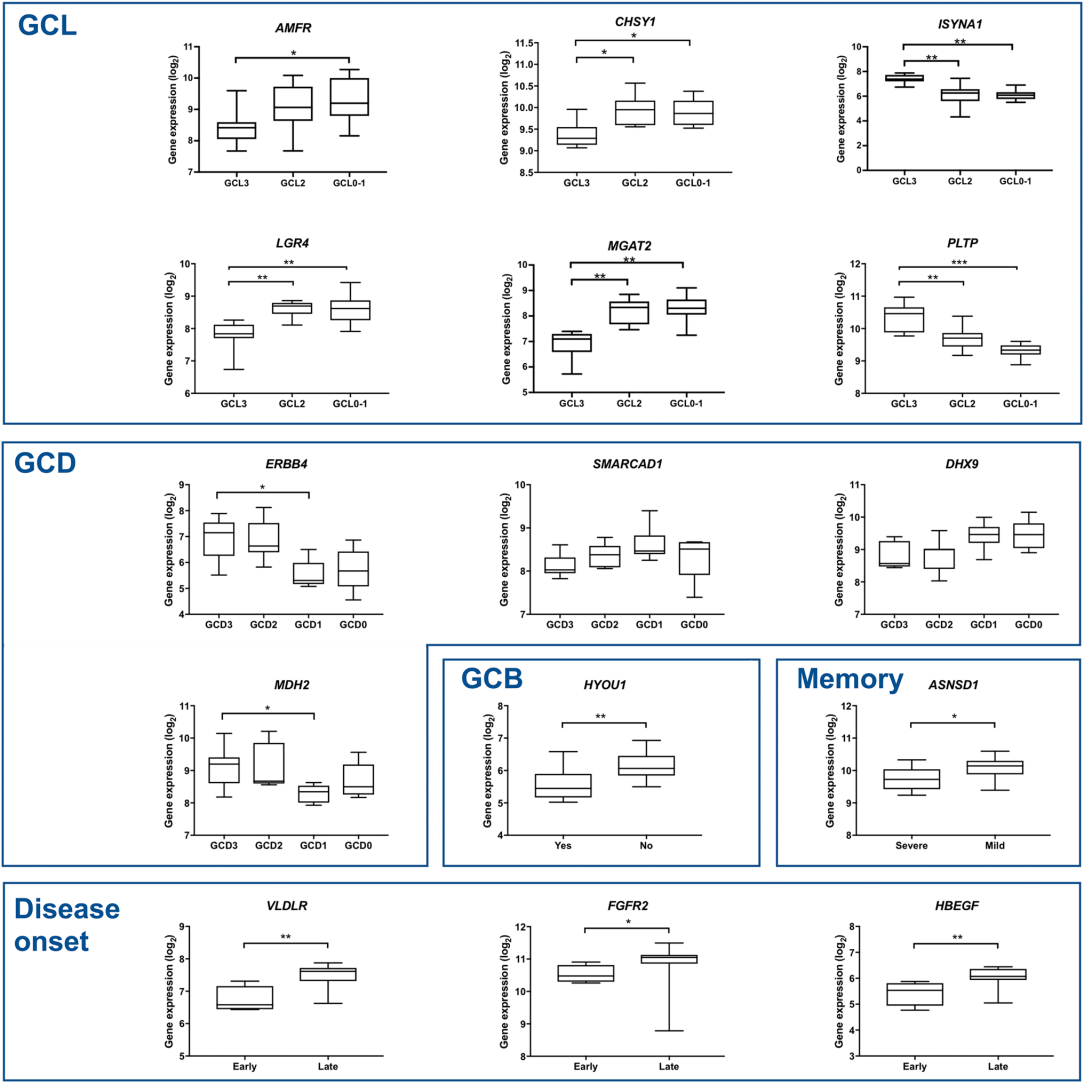
Gene expression plots for the HGS genes highly associated with specific histopathological or clinical data. The abbreviations-GCL, GCD, GCB-refer to histopathological alterations, as described in the Results and Material and Methods sections. Statistical significance values: *P < 0.05; **P < 0.005.

In the black and purple modules the HH genes are negatively correlated with GCL3 and GCD2, respectively. The black module harbors the iHub *MGAT2*—also an HGS gene -that controls N-glycan branching and is required for normal neuronal development and viability^51^. This gene is markedly hypo-expressed in the GCL3 hippocampi (Fig. 6). In the purple module it is worth mentioning the HHub *SDK2*, which encodes a sidekick molecule that mediates neuronal cell–cell adhesion^52^.

The red module has six HH genes, all involved in relevant brain and neuronal functions. Four of these genes are positively correlated with E-onset (Fig. 5): the HHub *OLFM1* codes for olfactomedin and promotes hippocampal axon growth after axonal injury^53^; the iHub *ST8SIA3* is involved in mediating sialylation and neuronal plasticity^54^, and increased polysialylation and neuronal circuitry remodeling have been reported in the hippocampus and entorhinal cortex of RMTLE patients^55^; the iHub, *SVOP*, codes for a synaptic vesicle transport protein structurally related to SV2A, the target of the anti-epileptic drug Levetiracetam^56,57^; the eHub *NCDN* encodes neurochondrin (norbin), which promotes neurite outgrowth, regulates hippocampal neurogenesis, modulates the metabotropic glutamate receptor 5, and was found to be downregulated in the hippocampus of intractable TLE patients^58^. Regarding the two negatively correlated genes: the eHub *ERBB3* mediates hippocampal neurogenesis through neuregulin-1/ERBB3 signaling, an essential process for memory^59^; and the eHub *SYNJ2*, that encodes synaptojanin-2 and is involved in astrocyte morphology remodeling^60^, was shown to influence human cognitive abilities^61^.

#### Functional interpretation of HGS (High Gene Significance) genes

Genes presenting high GS and high Module Membership (MM) values are considered HGS genes (see Methods) in a module and are significantly correlated with a particular trait^24^. Gene-trait correlations and biological functions for the HGS genes appear in Table 2, and the corresponding statistical data is shown in Supplementary Table S6 online. The functional interpretation of HGS genes was based on GO and ad hoc annotations^62^. The gene expression plots for HGS genes, the relationships between gene expression values and traits, and the statistical significance of these relationships are displayed in Fig. 6.

**Table 2:** HGS genes identified in modules associated with at least one trait: GCL, GCD, GCB, with severe memory impairment, or early/late disease onset.

Trait	Gene	GO database: biological process; molecular function
**GCL**		
Blue	*AMFR*	protein ubiquitination; learning or memory
Black	CHSY1*	chondroitin sulfate biosynthetic process
Turquoise	*ISYNA1*	inositol biosynthetic process
Brown	***LGR4***	positive regulation of canonical Wnt signaling pathway
Black	MGAT2*	protein N-linked glycosylation via asparagine
Brown	*PLTP*	phospholipid transport
**GCD**		
Blue	*ERBB4*	protein tyrosine kinase activity
Brown	*SMARCAD1*	chromatin remodeling
Blue	*DHX9*	DNA helicase activity
Turquoise	*MDH2*	RNA binding
**GCB**		
Blue	*HYOU1*	cellular response to hypoxia
**Severe memory impairment**^**L**^		
Brown	*ASNSD1*	glutamine metabolic process; asparagine biosynthetic process
**Disease onset**		
Red	*VLDLR*	reelin-mediated signaling pathway
	*FGFR2*	fibroblast growth factor-activated receptor activity
	*HBEGF*	epidermal growth factor receptor signaling pathway

^L^Indicates LHS group; *Genes that presented highest GS and MM values; in bold genes that are also iHubs.

Six out of the 15 HGS genes are correlated with GCL (Table 2) and their expression levels show significant differences between GCL grades (Fig. 6). Two of these genes, *AMFR* (alias *GP78*) and *PTLP*, are involved in learning and memory. *AMFR* encodes a receptor of the neurotrophic factor neuroleukin and its hippocampal expression is correlated with learning and memory in animal models^63,64^. *PTLP* codes for the plasma phospholipid transfer protein, which exerts a neuroprotective role through its ability to deliver vitamin E to the brain, and PTLP-KO mice have impaired memory and learning capabilities^65^. In GCL3 sclerotic hippocampi *AMFR* expression is increased from GCL3 to GCL0-1 and *PTLP* expression—neuroprotective—is decreased (Fig. 6). Two other genes, *CHSY1* and *ISYNA1* (alias *INOS*), are involved in the biosynthesis of chondroitin sulfate and inositol, respectively. Chondroitin sulfate is important for the hippocampal volume maintenance in TLE patients^66^. Persistent elevated inositol levels were found in the hippocampus of rats presenting repeated seizures after spontaneous seizure induction^67^, and are associated with pharmacoresistance in human TLE^68^. *ISYNA1* expression is decreased from GCL3 to GCL0-1, and *CHSY1*expression is increased. Finally, *LGR4* and *MGAT2*, which are also HH genes (Fig. 5), are relevant for brain and neuronal functioning. *LGR4* is a positive regulator of the canonical Wnt/β catenin signaling, which is disrupted both in the acute and chronic phases of TLE^48,69^. *MGAT2* is required for the normal assembly of complex Asn-linked glycans, being essential for normal neurological development^51^. The expression of these two genes is decreased in the sclerotic hippocampi with GCL3.

Four HGS genes are correlated with GCD (Fig. 6). Two of these genes—*ERBB4* and *MHD2-*are directly involved in epilepsy and have increased expression in GCD2/GCD3. *MHD2* codes for an RNA binding protein involved in the post-transcriptional downregulation of the sodium channel *SNC1A* expression in the hippocampus under seizure conditions^70^. Interestingly, epilepsy, hippocampal sclerosis, and febrile seizures are linked by common genetic variation around *SCN1A*^71^, The downregulation of *SCN1A* was also reported as an epilepsy biomarker by Guelfi et al.^20^, who studied the transcriptome of cortical tissue samples from 86 RMTLE patients. *ERBB4* codes for an important receptor of Neuregulin 1 (NRG1) and is involved in neurodevelopment and synaptic plasticity^72^. Both NGR1 and ERBB4 have been implicated in animal models and human epilepsy and recent experimental data showed that these two proteins are overexpressed in symptomatic epilepsy, suggesting that the NRG1-ERBB4 signaling probably act as a homeostasis modulator protecting brain from aggravated epileptiform activity^73^. The other two genes correlated with GCD are *SMARCAD1* and *DHX9*, whose average gene expression levels vary across GCD grades (Fig. 6; Supplementary Table S6 online). *SMARCAD1* is a chromatin remodeling gene acting in mammal adult neurogenesis^74^, while *DHX9* codes for a DEAH-containing helicase which regulates DNA replication, transcription, translation, microRNA biogenesis, RNA processing and transport, and maintenance of genomic stability^75^.

*HYOU1* is the only HGS gene correlated with GCB. Its overexpression prevents endoplasmic reticulum (ER) stress and rescues neurodegeneration^76^. ER stress has been recognized as a relevant etiological factor contributing to epilepsy-induced neuronal damage^77^. The expression of this gene is decreased in the sclerotic hippocampi with GCB.

The HGS gene *ASNSD1* is involved in asparagine biosynthesis and its expression is decreased in our patients with severe memory impairment. Asparagine synthesis is essential for brain development and asparagine deficiency has been associated with deficits in learning and memory^78^.

Three HGS genes are correlated with disease onset and have diminished expression in our early-disease onset patients. Interestingly, these genes belong to the red module (Table 2) and are involved in synaptic plasticity and memory processes. *VLDR* codes for a component of the Reelin pathway regulating neuronal migration and synaptic plasticity in the hippocampus^79^. *FGFR2* codes for the fibroblast growth factor receptor 2 and plays a role in the hippocampal short-term learning and long-term reference memory^80^, and *HBEGF* codes for the heparin-binding EGF-like growth factor and plays a significant role in hippocampal synaptic plasticity and memory formation^81^.

## Discussion

Module-trait correlation analyses revealed two meta-modules presenting high positive or negative correlations with severe (GCL3, GCD2, E-IPI, SMI, and SEFI) or mild (GCL0-1 and GCD1) phenotypes. In the meta-module I the transcriptional modules are positively correlated with the severe phenotypes and negatively with the mild phenotypes. Inversely, in the meta-module II all modules are negatively correlated with the severe phenotypes and positively with the mild phenotypes (Fig. 2c), except the red module, the only module that is positively correlated with E-onset and severe memory impairment. These correlations clearly indicate two distinct CA3 transcriptome profiles: one for severe and another for mild phenotypes. In addition, the functional enrichment analysis showed that the red and blue modules had many genes over-represented in neuron/excitability processes, such as neuron, glia, axon, myelination, excitability, and synapse (Fig. 3). In order to get a better understanding of how these modules are associated with DG alterations, cognitive impairment, and disease onset, we focused on the hierarchical characterization and functional interpretation of the genes within each module.

The blue and yellow modules (Fig. 4) harbor HH genes positively associated with pathophysiological mechanisms in epilepsy—such as vascular remodeling (*ITGB8, UCQRB*), glutamatergic excitatory system (*TANC2*, *SCLC25A18*) and reactive gliosis (*SPOCK1*)-and with putative compensatory mechanisms linked to hippocampal neuron survival (*KIF1B, YAP1*). In the turquoise module seven out of its nine HH genes are positively correlated with the mild phenotype GCL0-1 (Fig. 5). Two HH genes in this module, *PIK3C3* and *DCP1B*, are respectively involved in CNS neuronal homeostasis and in regulating human memory performance, thus indicating protective and compensatory mechanisms. Significantly, all above-mentioned genes, except for *SPOCK1*, are HHubs or eHubs i.e., genes with high intramodular and/or whole network connectivity. These genes are probably essential genes with a relevant role in defining the modules’ biological function^25,28^, namely, the association of blue and turquoise modules with severe and mild phenotypes, respectively.

The red module, occupying a separate branch in the eigengene dendrogram, is highly and positively correlated with E-onset, and positively correlated with severe memory impairment (Fig. 2c). Early MTLE onset with HS has a large impact on brain plasticity and on brain connectivity and memory^82,83^. Moreover, early seizure onset is also a predictive factor for pharmacoresistancy^6,84^, being associated with a more severe functional abnormality in the ictal hippocampus^83^. Interestingly, five out of the six HH genes in the red module are involved in processes compatible with early tissue damage and subsequent microstructural reorganization, specifically: axon sprouting (*OLFM1)*; neuronal circuitry remodeling (*ST8SIA3*); hippocampal neurogenesis, (*NCDN* and *ERBB3*), and astrocyte morphology remodeling (*SYNJ2*). Interestingly, the two hypo-expressed HH genes in the red-module, *ERBB3* and *SYNJ2*, are also related to cognitive abilities and memory. Therefore, the co-expression profile, functional role, and connective hierarchy of the HH genes in the red module (Fig. 5) appear to be compatible with the module’s phenotypic profile.

Contrariwise to hubs, that occupy a topologically central position in the modules and confer robustness to the co-expression network, HGS genes are usually at the network’s periphery and their expression shows significant variation across trait groups^25,28,85^. The HGS genes are significant for the traits, as described in the Results section and depicted in Fig. 6, and here we show how these genes may serve as biomarkers or therapeutic targets for RMTLE.

Let us consider firstly the six HGS genes correlated with GCL. We found that *ISYNA1*, whose brain expression is persistently elevated in a rat model of epilepsy^67^, is more expressed in GCL3 than in GCL2 and GCL0-1, confirming its proposed role as RMTLE biomarker and its potential as a therapeutic target^67,86^. *CHSY1* is related to the maintenance of hippocampal volume in RMTLE patients and its expression increases from GCL3 to GCL0-1, so indicating a compensatory mechanism and the gene’s usefulness as biomarker. The genes *AMFR* and *PTLP* are respectively involved in neuronal survival and in neuroprotection, and both are necessary for memory and learning, but *AMFR* expression increases from GCL3 to GCL0-1 whereas the *PTLP* expression decreases, thus indicating pathogenic and compensatory mechanisms. Finally, *LGR4* and *MGAT2*, also HH genes and respectively involved in regulating Wnt/β catenin signaling and neuronal viability, showed increased expression levels from GCL3 to GCL0-1, implying a compensatory mechanism. These two genes may be meaningful biomarkers for RMTLE and hippocampal sclerosis.

Four HGS genes are correlated with GCD and two of them, *ERBB4* and *MDH2*, directly involved in epileptic processes, have significantly higher expression in GCD3 and GCD2, being potential biomarker candidates. The other two genes, *SMARCAD1* and *DHX9*, are involved in chromatin remodeling and maintenance of genomic stability, and their average expression levels varied across GCD grades. The HGS gene *HYOU1* has a neuroprotective role and, as expected, is relatively higher expressed in the absence of GCB. *ASNSD1*, a gene involved in biological processes related to memory, showed diminished expression in patients with severe memory impairment. Finally, three HGS genes, *VLDR*, *FGFR2*, and *HBEGF*, that are involved in synaptic plasticity and memory formation and belong to the red module—which is correlated with E-onset and memory impairment-showed significantly decreased expression in early disease onset patients.

Lastly, our results show that the histogenomic approach adopted here is advantageous for investigating the cellular and molecular mechanisms underlying RMTLE, although a few limitations need to be considered. Firstly, it only allows to obtain the transcript expression profiles and histopathological features at the time of surgery. Secondly, there is no availability of control tissue, since the use of autopsy material, or brain tumor resected tissue, as control is not either feasible or technically adequate^87^. In order to circumvent these limitations several groups^88,89^ including our own^90^, have recurred to animal models of acquired epilepsy for conducting temporal analyses of brain histological and molecular changes that arise after an initial precipitating injury.

In conclusion, by adopting a systems biology approach and integrating clinical, histopathological and transcriptomic data, we were able to identify transcriptional modules highly correlated with DG alterations, cognitive dysfunctions, and disease onset in our RMTLE patients. The functional characterization of the high-hierarchy genes in each module allowed us to unveil the modules’ main biological functions, paving the way for further investigations on their roles in RMTLE pathophysiology. Moreover, we found several HGS genes that may potentially become novel biomarkers and/or therapeutic targets in RMTLE. These results are relevant considering the urge for identifying the genomic mechanisms underlying RMTLE, what could lead to more effective therapeutic interventions.

## Material and methods

### Patients and brain tissue specimens

A total of 57 RMTLE patients (aged 18–55 years) with unilateral mesial temporal sclerosis (MTS) who underwent corticoamygdalohippocampectomy were included in this study, which was approved by the research ethics committees of Hospital das Clínicas da FMUSP and Hospital Albert Einstein, São Paulo, SP, Brazil, under numbers 251/05 and CAEE 0122.0.028.174.05. A written informed consent was obtained from all patients. All patients were diagnosed according to ILAE criteria^91^ and underwent preoperative clinical, video-EEG monitoring, neuropsychological and neuroimaging evaluations. The inclusion criteria were as follows: unilateral TLE refractory to two or more antiepileptic drugs; focal or focal-to-bilateral seizures; eight or more years of education; IQ superior to 70; right handedness. None of the patients had extratemporal epileptiform activity, other encephalic lesions on MRI except TLE, intellectual disability, first-degree family members with epilepsy or with a history of a febrile seizure. Tissue explants from CA3 were obtained at surgery room and immediately processed for global gene expression analysis. All the resected hippocampal specimens were analyzed by histopathology. Demographic, clinical, histological and cognitive data of all patients are summarized in Supplementary Table S1 online. All methods and experiments were carried out in accordance with relevant guidelines and regulations.

### Cognitive function evaluation

A total of 44 patients and 60 healthy volunteers (Supplementary Table S2 online) underwent cognitive function evaluation. They were from the same socioeconomic and cultural background. Volunteers were aged 18–55 years and had no central nervous system disorders or other medical disorders affecting cognitive abilities. All were right-handed. Patients and controls with IQ score > 70, eight years of education or more, and without major psychiatric comorbidities, underwent a cognitive test battery that included: (i) Rey auditory verbal (RAVLT) and visual design (RVDLT) learning test, Rey-Osterrieth complex figure test (ROCF), facial recognition, and logical memory, for memory; (ii) semantic and phonological fluency, word comprehension, and object, verbal, proper noun and responsive naming; and (iii) Stroop test (time and error scores); Digit Span test, phonemic verbal fluency (FAS test), and Wisconsin test (WCST), for executive function (Supplementary Table S3 online). The collected data were analyzed using the Receiver Operating Curve (ROC) analysis, comparing patients’ and controls’ performances to yield best sensitivity and specificity, in order to establish a cut-off score for each test (Supplementary Table S3 online). We classified patients as normal or impaired, compared with controls, using Mann–Whitney test for each test. Values of *P* < 0.05 were considered as statistically significant. We generated a memory, language and executive function impairment score. This score was based on the number of tests for each cognitive domain (memory, language, and executive function).

### Histological analysis

Histological analysis was performed in 43 resected hippocampi (Supplementary Table S1 online). Histological processing was undertaken as previously described^92^. Surgical specimens were fixed in 4% paraformaldehyde (PFA) in 0.1 M phosphate buffered saline (PBS) for 24 h and then transferred to a solution of 1% PFA plus sucrose 30% in PBS 0.1 M for one week. Each hippocampus (25–30 mm length) was carefully oriented, trimmed and sectioned in the plane perpendicular to its longitudinal axis. The entire hippocampus (head and body) was used in the present investigation (50–60 slices/patient). Because sections cut tangentially to the principal cell layers or at inconsistent angles from the longitudinal axis of the hippocampus can produce unusual histological features, considerable care was taken to ensure that all hippocampal sections were cut in a plane strictly perpendicular to the longitudinal axis of the hippocampus. Sixty- micron coronal slices through the entire extension of the hippocampus were obtained using a cryostat (− 21 °C). One out five slices (e.g., the 1^st^, 6^th^, 11^th^ and so forth) were mounted on gelatin-coated slides and stained with cresyl violet (Nissl).

The type of HS was described according to Blümcke et al.^27^ as HS ILAE type 1 (severe CA1 and CA4 neuronal loss and gliosis), HS ILAE type 2 (CA1 neuronal loss and gliosis), HS ILAE type 3 (CA4 neuronal loss and gliosis) or no-HS (preserved cell density and gliosis only), based on light microscopy of the hippocampal subfields, using a 5 × objective lens (DM750, Leica Microsystems, Germany) throughout the entire hippocampus. Only sections presenting the three CA subfields were included.

Semiquantitative assessment focused on dentate gyrus abnormalities, particularly cytoarchitectural disorganization (loss, dispersion and bilamination) in four non-adjacent hippocampal body slices. Granule cell loss (GCL) was defined as areas of reduced granule cell layer width, reduced neuronal cell density or granule cell layer disruption. GCL was graded in three categories: grade zero to 1-no GCL to mild neuronal density reduction, grade 2-moderate neuronal density reduction, grade 3-severe neuronal density reduction. Granule cell dispersion (GCD) was defined as broadening of the granule cell layer and loss of normal boundaries. GCD was graded into four subjective categories: grade zero—no GCD, grade 1-mild granule cell layer broadening with few ectopic granule cells in the inner molecular layer, grade 2-moderate granule cell layer broadening with ectopic granule cells beyond the inner molecular layer, and grade 3-extensive granule cell layer broadening invading the outer molecular cell layer. Granule cell bilamination (GCB) was described as present or absent. Both ectopic clusters of granule cells in the molecular layer (focal GCB) and complete duplication of granule cell layer (diffuse GCB) were considered. Due to possible artifacts, we did not evaluate areas at angles, end points of the C-shaped granule cell layer, or blood vessels vicinities. To ensure reproducibility, the histological analysis was carried out by two different experienced pathologists following the same protocol. Overall, the observations were concordant. Eventual disagreements were discussed until consensus was found.

### RNA extraction

Fresh explants from hippocampal CA3 region (3–4 mm^3^) from 22 RMTLE patients (Supplementary Table S1 online) were obtained at the operating room and immediately preserved with RNA*later* (Qiagen cat. no. 76106, Valencia, CA). Neuropathology analysis of the resected hippocampi confirmed that the CA3 explants were obtained at the proper site^92^. The preserved explants were homogenized with TissueRupter (Qiagen, cat. no. 9001272 Valencia, CA) and total RNA was extracted from the homogenates using the RNeasy Lipid Tissue Kit (Qiagen cat. no. 74804, Valencia, CA) according to the manufacturer’s instructions. RNA quality was assessed on the Agilent BioAnalyzer 2100 (Agilent, Santa Clara, CA). All samples were stored at -80 °C until used in hybridization experiments.

### Microarray hybridization and gene expression analysis

In order to determine gene expression profiles, 4 × 44 K DNA microarrays (Whole Human Genome Microarray Kit, Agilent Technologies, cat no. G4112F, Santa Clara, CA) were used. The procedures for hybridization using the fluorescent dye Cy3 followed the manufacturer’s protocols (One-Color Microarray-Based Gene Expression Analysis—Quick Amp Labeling). The images were captured by the reader Agilent Bundle according to the parameters recommended for bioarrays and extracted by Agilent Feature Extraction software version 9.5.3. (https://www.agilent.com/). Spots with two or more flags (low intensity, saturation, controls, etc.) were considered as NA, that is, without valid expression value. An in-house algorithm in R environment (version 3.4.4^93^) was used for excluding transcript spots presenting one or more NAs and for converting gene expression values to log base 2. Through this procedure we identified 8,104 Gene Ontology (GO) annotated genes. Data normalization was performed using limma package^94,95^ in R environment (version 3.4.4^93^). All microarray raw data have been deposited in GEO public database (http://www.ncbi.nlm.nih.gov/geo), a MIAME compliant database, under accession number GSE163296.

### Weighted Gene Co-expression Network Analysis (WGCNA)

The normalized gene expression data was used for WGCNA. Network was constructed using the WGCNA package^24^ (version 1.69–81; https://horvath.genetics.ucla.edu/html/CoexpressionNetwork/Rpackages/WGCNA/) in R environment (version 3.4.4^93^). Pearson’s correlation coefficient was used for obtaining gene co-expression similarity measures and for the subsequent construction of an adjacency matrix using soft power and topological overlap matrix (TOM). Soft-thresholding process transforms the correlation matrix to mimic the scale-free topology. TOM is used to filter weak connections during network construction. Module identification is based on TOM and in average linkage hierarchical clustering. Keeping to the scale-free topology criterion, soft power β = 12 (R^2^ = 0.910) was considered (Supplementary Fig. S5 online). Finally, dynamic tree cut algorithm was used for dendrogram’s branch selection. The module eigengene (ME) is defined as the first principal component of a given module, which can be considered a representative of the gene expression profiles in a module. Module Membership (MM), also known as eigengene-based connectivity (kME), is defined as the correlation of each gene expression profile with the module eigengene of a given module.

#### Module-trait association

Firstly, we obtained the gene significance (GS), i.e. a value for the correlation between specific traits (Supplementary Table S1 online) and gene expression profiles^24^. This analysis was conducted for three different trait matrices. One matrix for demographic, clinical, and histopathological data of the 22 TLE patients and the two other matrices for cognitive features: (i) one for the left HS patients (n = 14), and (ii) another for right HS patients (n = 8). The average GS value for a particular module is considered as a measure of module significance (MS). The GS values were obtained using Pearson’s correlation and, for assigning a *P*-value to the module significance, we used the Student’s t test. The modules presenting high positive or negative correlation values (r ≥|0. 50| and *P* < 0.05) with a trait were selected for functional analysis. Modular gene set enrichment analysis for GO Biological Process (BP) terms was accomplished by using the Enrichr online web-based tool^29^.

#### Node categorization

Intramodular node connectivity was calculated considering: (i) *k*_Total_, the whole network connectivity of each gene; (ii) *k*_Within_, gene connections with other genes in the same module^24^. Genes presenting high *k*_Total_ and *k*_Within_ are classified as high hubs (HHubs), genes presenting high *k*_Total_ but low *k*_Within_ are called eHubs, and genes presenting high *k*_Within_ but low *k k*_Total_ are the iHubs. We plotted all gene values in a *k*_Total_ vs. *k*_Within_ graphic (Supplementary Figs. S3 and S4 online). Additionally, the differential gene expression profile was assessed through GS values, ie. the GS of the *n*^*th*^ gene is the correlation measure between the *n*^*th*^ gene expression and the specific trait. Positive or negative GS values mean that the *n*^*th*^ gene is hyper- or hypo-expressed for the specific trait.

#### Genes presenting high gene significance (GS) values (HGS genes)

The Module Membership (MM), i.e. a measure of intramodular connectivity^24^, and the GS values were used for candidate gene marker identification. Genes presenting high GS and high MM were considered as HGS genes in the module and significantly associated with at least one trait. This analysis was conducted for left and right sides separately. We plotted all gene values in a MM (x-axis) vs. GS graphic (y-axis).

### Clinical and pathological correlation analyses

Clinical and pathological data from 43 patients and respective hippocampal samples (Supplementary Table S1 online) were used for correlation analyses between a pair of selected phenotypic variables, e.g. GCL grade vs. memory impairment score for left-HS patients, IPI vs. HS side, HS vs. language impairment, etc. Comparison groups were set for three data matrices: (i) all patients; (ii) LHS group, including left-HS patients only; and (iii) RHS group, including right HS patients only. These analyses were conducted using the Spearman test and the *p* value < 0.05 was considered significant.

## Supplementary Information

Supplementary Information 1.

Supplementary Information 2.

## Data Availability

The datasets generated during and/or analyzed during the current study are available from the corresponding author on reasonable request.
