# Supplementary material for: Hippocampal CA3 transcriptional modules associated with granule cell alterations and cognitive impairment in refractory mesial temporal lobe epilepsy patients
Source: Sci Rep. 2021 May 13;11:10257. doi: 10.1038/s41598-021-89802-3 (PMC8119682; doi:10.1038/s41598-021-89802-3)
Supplement: Supplementary file 1 — Supplementary Information 1. [file 41598_2021_89802_MOESM1_ESM.pdf]

Hippocampal CA3 Transcriptional Modules Associated with Granule Cell Alterations and Cognitive Impairment in Refractory Mesial Temporal Lobe Epilepsy Patients

Silvia Yumi Bando[1#]; Fernanda Bernardi Bertonha[1#]; Luciana Ramalho Pimentel-Silva[2]; João Gabriel Mansano de Oliveira[3]; Marco Antonio Duarte Carneiro[3]; Mariana Hiromi Manoel Oku[3]; Hung-Tzu Wen[4]; Luiz Henrique Martins Castro[3]; Carlos Alberto Moreira-Filho[1*].

[1]Department of Pediatrics, Faculdade de Medicina da Universidade de São Paulo, São Paulo, SP, Brazil.
[2]Department of Neurology, Faculdade de Ciências Médicas da Universidade Estadual de Campinas, UNICAMP, Campinas, SP, Brazil.
[3]Department of Neurology, Faculdade de Medicina da Universidade de São Paulo, São Paulo, SP, Brazil.
[4]Epilepsy Surgery Group, Hospital das Clínicas da FMUSP, São Paulo, SP, Brazil.

[#]*These authors contributed equally to this work.*

[*]*Corresponding author*
Prof. Dr. Carlos Alberto Moreira-Filho
Departament of Pediatrics, Faculdade de Medicina da Universidade de São Paulo
Av. Dr. Enéas Carvalho Aguiar, 647
05403-000, São Paulo, SP, Brazil
E-mail: carlos.moreira@hc.fm.usp.br

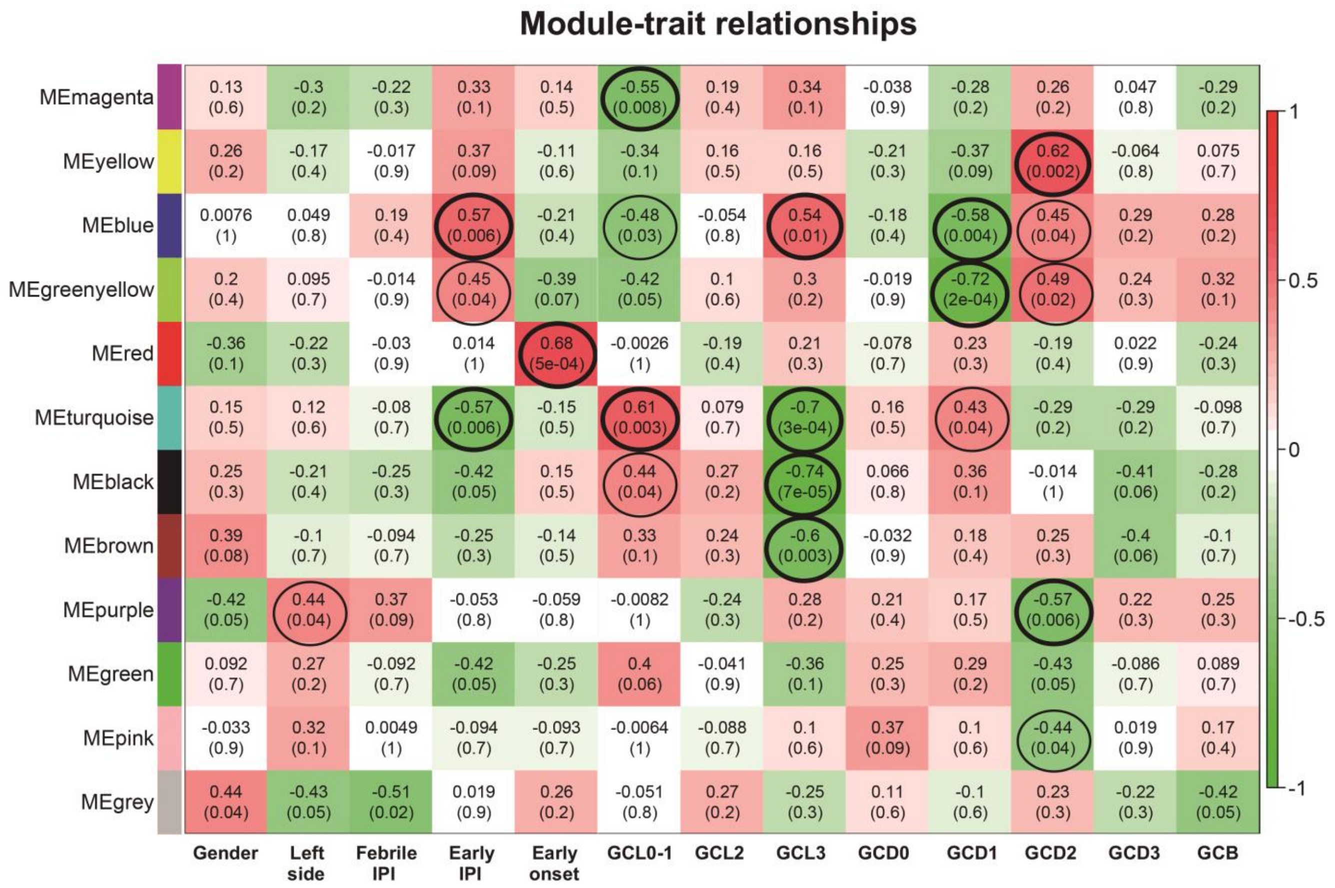

Supplementary Figure S1. **WGCNA modules and Module Eigengene (ME) correlations with demographic, clinical, and histological data (traits).** In the rows, MEs are named by their module colors. In the columns are shown the traits of interest. Numbers inside each colored box are the correlation coefficients between the ME and the specific trait, with p-value between round brackets. The more intense the color of the box, the more negatively (green) or positively (red) correlated is the module with the trait. Ellipse shaped objects show significant module-trait correlations ($p < 0.05$). Thick ellipse shapes depict for high correlation value ($r > |0.50|$).

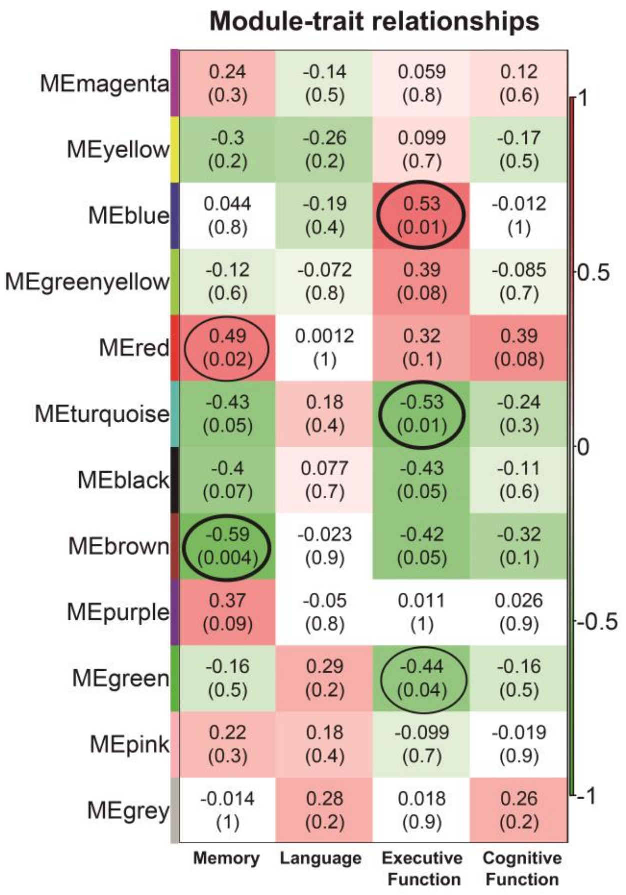

Supplementary Figure S2. **WGCNA modules and Module Eigengene (ME) correlations with cognitive function impairments.** In the rows, MEs are named by their module colors. In the columns are shown the traits of interest. Numbers inside each colored box are the correlation coefficients between the ME and the specific trait, with p-value between round brackets. The more intense the color of the box, the more negatively (green) or positively (red) correlated is the module with the trait. Ellipse shaped objects show significant module-trait correlations ($p < 0.05$). Thick ellipse shapes depict high correlation values ($r > |0.50|$).

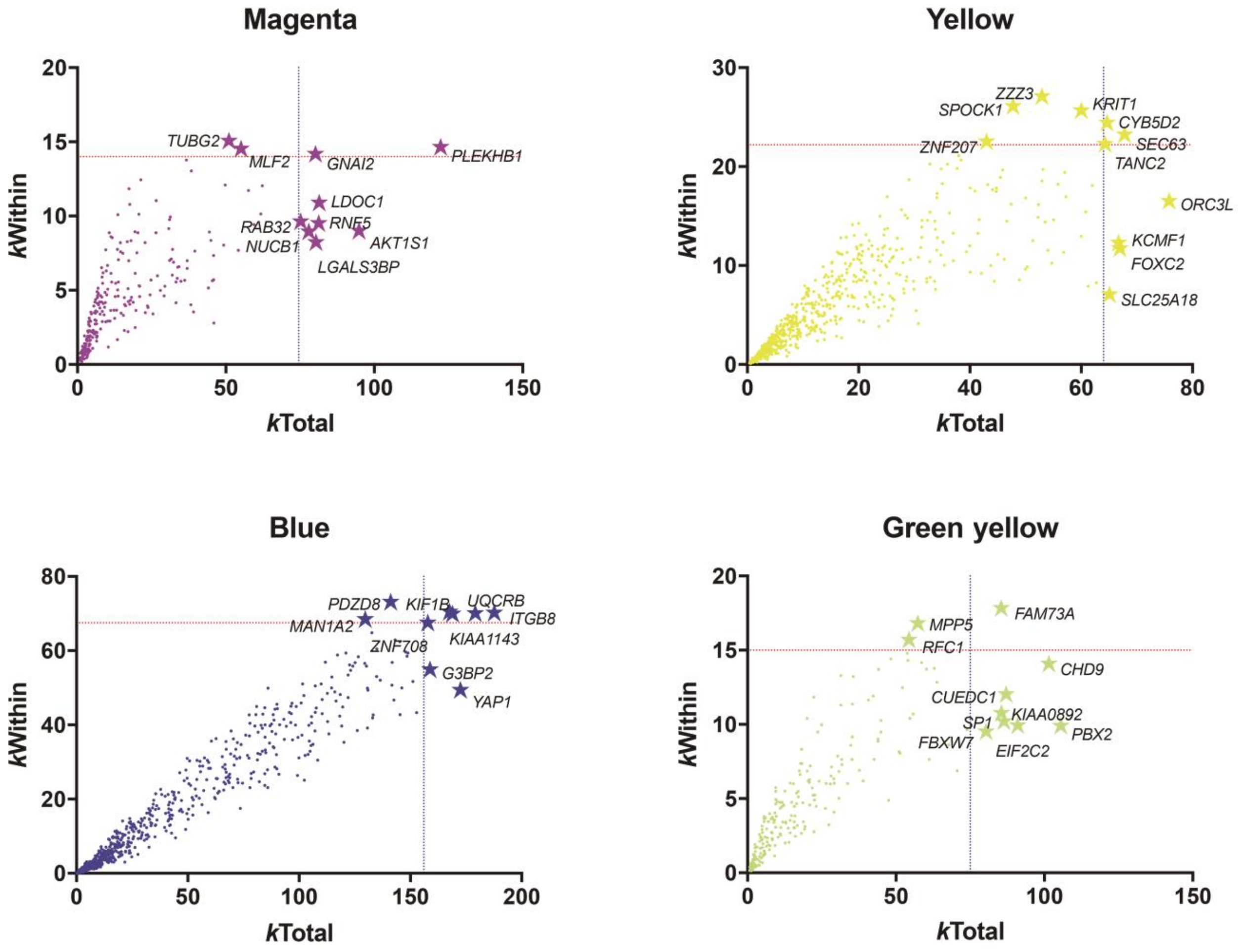

Supplementary Figure S3. **Intramodular node categorization for meta-module I.** k_{Total} vs. k_{Within} plots for the modules belonging to meta-module I - magenta, yellow, blue, and green yellow. High hierarchy (HH) genes are identified by colored star shapes and their respective gene symbols.

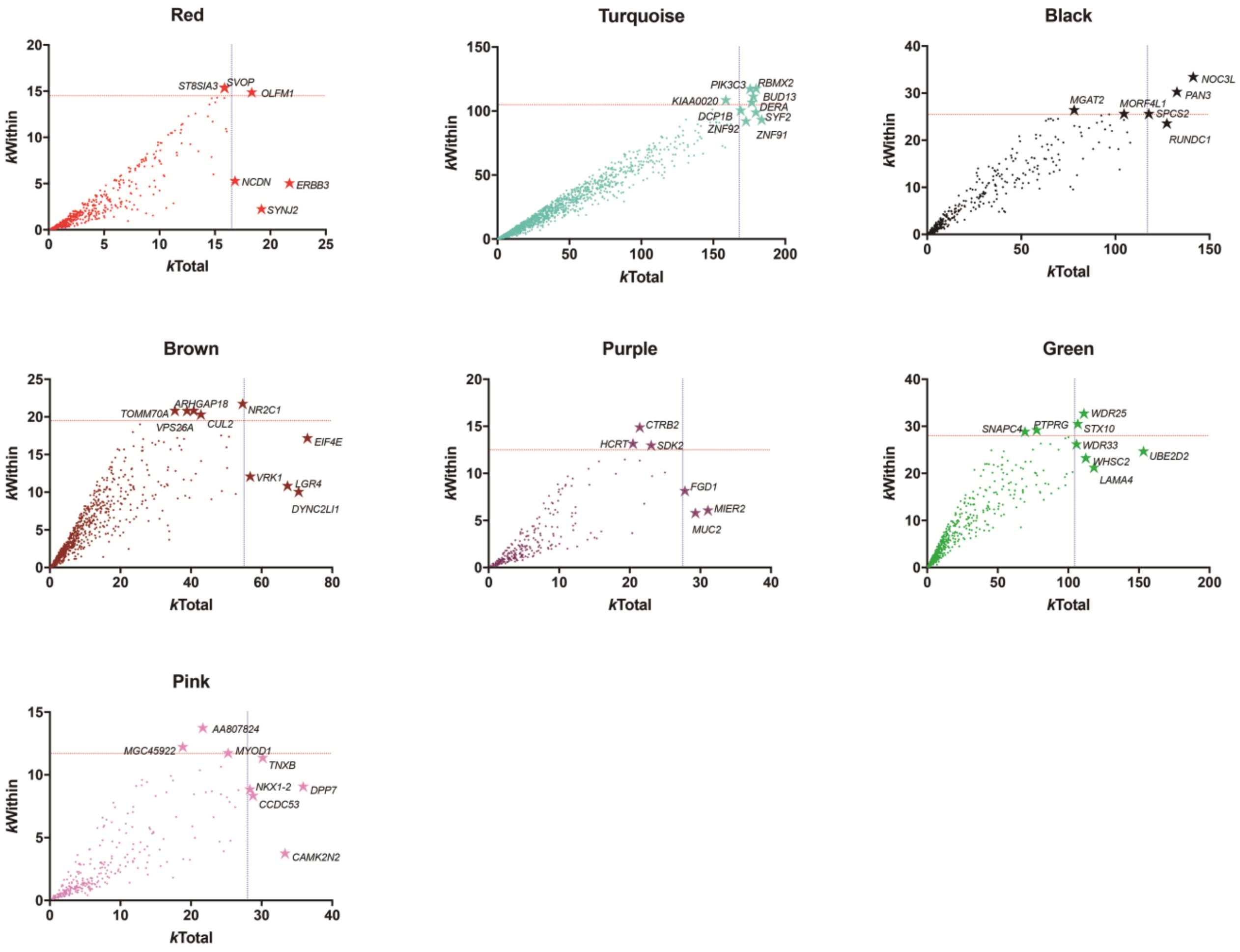

Supplementary Figure S4. **Intramodular node categorization for meta-module II.** k_{Total} vs. k_{Within} plots for the modules belonging to meta-module II – red, turquoise, black, brown, purple, green, and pink. High hierarchy (HH) genes are identified by colored star shapes and their respective gene symbols.

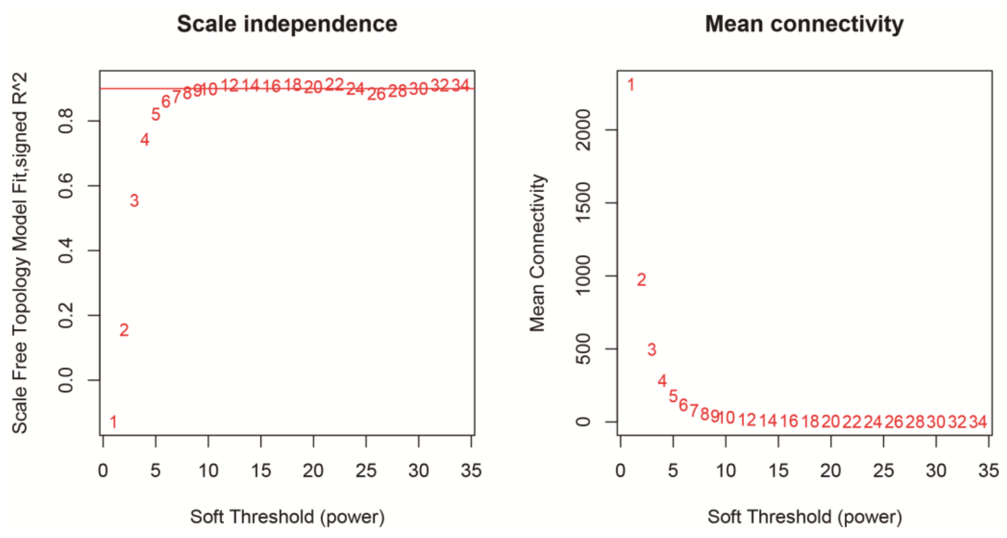

Supplementary Figure S5. **Selection of the soft-thresholding power (β)**. The dataset was fit to a scale-free model of proposed values for β ranging from 1 to 35 (numbers inside the plots). Approximate scale-free topology is attained around soft-thresholding power of 12, which reflects the inflection point where model fit begins to decrease with power increasing (left panel). The right panel shows the plot of the mean connectivity along with the soft-thresholding power. The red line indicates the scale-free topology R^2 fit index cut-off of 0.910.
